# The impact of pension on the health behavior of elderly rural residents: evidence from China

**DOI:** 10.1186/s12877-024-04783-y

**Published:** 2024-03-18

**Authors:** Rui Li, Duanyang Gao, Yuying Yang

**Affiliations:** 1 College of Public Administration and Humanities, Dalian Maritime University, Dalian, 116026 China; 2 School of Sociology and Population Studies, Renmin University of China, Beijing, 100872 China; 3 School of Agricultural Economics and Rural Development, Renmin University of China, Beijing, 100872 China

**Keywords:** Health behavior, Pension, Rural elderly residents, Regression discontinuity, China

## Abstract

**Background:**

Unhealthy behavior is an important factor threatening the health of older rural residents in China. We examine the effects of receiving pension on elderly rural residents’ health behavior (namely conscious control of sugar, salt, and edible oil intake, as well as learning health or wellness knowledge), also including effect heterogeneity by income level and gender.

**Methods:**

Using China Rural Revitalization Survey (CRRS) in 2020, we used the policy rule of the China’s New Rural Pension Scheme that only those people who are over 60 years old can have access to pension as the natural experiment, and explore the causal effect of receiving pensions on the health behaviors by using regression discontinuity design method.

**Results:**

Having access to pension can improve the health behavior of rural elderly residents, including increasing the probability of rural residents’ conscious control of sugar (*p* < 0.1) and conscious control of salt intake (*p* < 0.1), which is still valid after a series of robustness tests. Heterogeneity analysis finds that access to pensions is more likely to improve the health behavior of low-income families and male rural elderly residents.

**Conclusions:**

The research has expanded the discussion on the correlation between the pension and the health of rural elderly residents, and the conclusion provides important policy implications for optimizing the rural elderly insurance system and leveraging pension to improve the health behavior of rural elderly residents.

## Introduction

China is the country with the largest elderly population in the world and one of the countries with the fastest aging rate. “Outline of the Healthy China 2030 Plan” points out that, China will enter a “moderately aging society” (the proportion of the population aged 60 and above exceed 20% of the total population), with aging accompanied by declining cognitive, motor, sensory functions, as well as increasingly prominent health issues such as nutrition and psychology, bringing the challenge of poor health conditions for the elderly during 2021–2025 [1]. Compared with urban areas, the aging rate of rural population is faster and deeper; compared with urban elderly people, there is greater room for improvement in their physical health status in rural areas [2–4]. According to the China’s seventh national population census and the announcement on the development of China’s national elderly care in 2020, the proportion of elderly people aged 60 and above and 65 and above in rural China to the total rural population is 23.81 and 17.72%, respectively, which are 7.99 and 6.61% higher than those in urban areas, meeting the standard of a moderately aging society [5, 6].

Numerous studies have confirmed the correlation between elderly health status and healthy behavior. If the dietary taste is too strong, it can lead to chronic disease comorbidities in the elderly [7, 8], and health education can significantly improve the healthy behavior and self-care ability of the elderly [9, 10]. Research also suggests that elderly people develop a light diet habit, reduce oil intake, engage in active physical activities, and get moderate sleep [11, 12]. Due to limited economic conditions and low educational levels, the dietary habits and structure of rural elderly people are not ideal enough [13, 14]. In recent years, the health knowledge level of Chinese residents has been greatly improved, but their daily behavior health level is still at a relatively low level [15, 16]. The theory of social ecology points out that the macro system of national policies can have a profound impact on individual behavioral choices [17–19]. Therefore, how to leverage the government’s supportive role [20], through policy intervention in health behavior, to achieve health improvement for elderly people in rural areas and rural healthy aging is of utmost importance.

Previous research on the impact of pension or medical insurance on health behavior has mainly focused on examining the behavior of research subjects such as smoking, drinking, and exercising. Medical insurance increases the probability of insured individuals smoking and drinking alcohol [21, 22]. Through exploring the relationship between personal insurance choices and smoking, alcohol consumption, exercise, and obesity, researchers finds that medical insurance increases adverse health behaviors among insured individuals [23, 24]. In terms of Chinese research, using China Health and Nutrition Survey (CHNS), scholars test whether there are moral hazard in advance in the basic medical insurance, and find that participation in the basic medical insurance would not lead to an increase in smoking and drinking behavior [25, 26]. Based on China Family Panel Studies (CFPS) 2012 data, a study uses smoking and alcohol consumption as proxy variables for health behavior, exploring the impact of China’s New Rural Pension Scheme on the health behavior of rural elderly people. The results show that pension reduce the probability of recent smoking and daily smoking among elderly people, but do not have a significant impact on their drinking behavior [27]. Basing on China Health and Retirement Longitudinal Study (CHARLS) 2011 and 2013 data, research uses three indicators as proxy variables for health behavior, namely, smoking, alcohol consumption and frequent exercise. The finds including that, the impact of medical insurance on smoking behavior is heterogeneous under self-assessment of health status, and the impact on alcohol consumption and exercise behavior is not affected by self-assessment of health level [28]. Utilizing tobacco expenditure, alcohol expenditure and household health expenditure as proxy variables for health behavior is also obtained research application [29].

It is significant to note that there are more diverse indicators for measuring healthy behavior. Health behavior has been defined as all behaviors and perceptions taken by individuals (families and society) to maintain or increase their health level to achieve self realization and satisfaction [30]. In terms of indicators measurement, scholars have also made many efforts, which including developing the Health Promoting Lifestyle Profile Nursing Research [31], measuring health behavior from six dimensions: nutritional behavior, health responsibility behavior, self actualization behavior, social support behavior, exercise behavior, and stress management behavior respectively [31], formulating indicators of healthy behavior, including sleep time, dietary patterns, physical activity, smoking and alcohol consumption, and physical health status [32]. On this basis, relating research compile health behavior to more specific 10 indicators, including life pattern, diet pattern, breakfast intake, interval intake, nutrition emphasis, salt control, nutrition balance, sleep time, exercise, drinking and smoking [33]. However, in the study of the impact of insurance on health behavior, rich health behavior measurement indicators have not been fully applied.

Unlike the previous studies, our research has the following contributions. Firstly, regarding measuring health behavior indicators, whether residents consciously control sugar, salt, and edible oil intake, learn health knowledge, or health preservation knowledge, indicators are selected as proxy variables for health behavior. However, the commonly used indicators for measuring health behaviors include smoking and alcohol consumption which can be more enriched. Daily cognition and related data also indicate that these two behaviors are more common among men. Therefore, the above shortcomings may affect the practical evaluation of residents’ pension insurance policies. Secondly, regarding research methods, using the regression discontinuity method to examine causal effects is a beneficial exploration of pension policy intervention in rural residents’ health behavior. Thirdly, we delve into how pension benefits can be more targeted in their design, such as how to address the preferences of vulnerable groups and how to tilt them to provide a reference for the institutional reform of rural pension insurance in China and other developing countries.

The New Rural Pension Scheme is a social pension insurance system organized and implemented by Chinese government to ensure the basic living conditions of rural residents in their old age, which has been piloted since 2009 and achieved full coverage by the end of 2012 [34]. In 2014, it merged with Urban Resident Pension Scheme to form Urban and Rural Resident Pension Scheme [35, 36]. For rural elderly residents, the economic benefits generated by this income cannot be ignored [37]. Chinese government emphasizes that health should be integrated into all policies, a comprehensive health impact assessment system should be established, and the impact of various economic and social development plans and policies, as well as major engineering projects on health should be systematically evaluated. Therefore, based on the quasi natural experiment that elderly people over 60 years old can receive resident pension, this article uses regression discontinuity and data from the China Rural Revitalization Survey (CRRS) to select more universal health behavior indicators for rural elderly residents, exploring the causal effect of pension on the health behavior of rural elderly residents, in providing new empirical evidence for promoting healthy aging in Chinese and other developing countries’ rural areas.

## Materials and methods

### Data

We used data from the China Rural Revitalization Survey (CRRS). CRRS is a nationwide large-scale rural tracking survey conducted by Rural Development Institute, Chinese Academy of Social Sciences. The research team conducts a tracking survey every 2 years. The first survey of farmers and villages was conducted in 2020, with face to face questionnaire survey conducted in 50 county (city) units and 156 township (town) units in 10 provinces (districts) across the country. In terms of sample selection, the research group comprehensively considered the level of economic development, regional location, and agricultural development situation and randomly selected sample provinces from the eastern, central, western, and northeastern regions. An equidistant random sampling method was used to select sample counties based on the per capita GDP at the county level in the province, and considered covering the entire province (region) as much as possible in space. Using the same sampling method, sample townships and villages were randomly selected based on their economic development level. Finally, randomly selected sample households were based on the roster provided by the village committee. The sample provinces specifically include Guangdong Province, Zhejiang Province, Shandong Province, Anhui Province, Henan Province, Heilongjiang Province, Guizhou Province, Sichuan Province, Shaanxi Province, and Ningxia Hui Autonomous Region.

A total of 15,554 samples were obtained from the face to face questionnaire survey. The content covers basic personal information, family situations, health status, and other information about farmers. This article selects the latest cross-sectional data released in 2020 for analysis (regression discontinuity design is locally random and does not require tracking data). Referring to existing research on the new rural social security pension [38], we selected samples aged 45 to 75 years old, excluded samples with missing covariates, and finally analyzes 5803 samples.

### Variable

The outcome variable of regression discontinuity is health behavior. Four questions were selected as proxy variables for health behavior, namely “Do you consciously control salt intake?”, “Do you consciously control sugar intake?”, “Do you consciously learn health or wellness knowledge?”, and “Do you consciously control edible oil intake?”. The answer “Yes” was assigned a value of 1, and “No” was assigned a value of 0. The disposal variable is whether to receive the new agricultural insurance pension. “Yes” is assigned a value of 1, and “No” is assigned a value of 0. In addition, as mentioned earlier, due to certain delays in pension payments, relevant studies have selected ages after 60 years old, such as 60.5 years old. However, due to the lack of specific research months in the CRRS, precise age cannot be obtained. Therefore, this article chooses 61 years old as the cutoff point (Cutoff).

In addition, in order to improve the accuracy of regression discontinuity estimation, this article also sets control variable (covariate) based on questionnaire questions, including gender (questionnaire options include male and female), education level (questionnaire options include not attending school, primary school, junior high school, high school, technical secondary school, vocational high school, college, and undergraduate), marriage (questionnaire options include married, unmarried, divorced, and widowed), BMI (questionnaire requires respondents to fill in their weight and height), disabled (questionnaire provides options for physical disability, brain damage/intellectual disability, visual impairment, hearing impairment, language impairment, and no disability), chronic diseases (questionnaire provides options for hypertension, dyslipidemia, blood glucose abnormalities, heart disease, language disorders, cancer or malignant tumors, liver or kidney or stomach diseases, neurological or psychiatric disorders, memory disorders, other chronic diseases, no chronic disease), and household income (questionnaire asks respondents in detail about the income of 8 major categories and and 23 subcategories of households, and summarizes them to obtain the household income. The 8 major categories of income include net income from planting, breeding, forestry, fishery, non-agricultural, wage/work income, property income, and transfer income) [39–43].

Table 1 summarizes the sample characteristics. The elderly individuals were divided into two groups based age, namely 45–61 years old (excluding 61 years old) and 61–75 years old (including 61 years old). There are 3816 samples in the 45–61 age group, with males accounting for 50.68%. In terms of education level, 48.45% had an educational level of junior high school, 40.75% had an educational level of primary school and below, 9.85% had an educational level of high school/technical secondary school/vocal high school, and only 0.94% had an educational level of college/undergraduate. 95.78% of people are married. The average BMI index is 26.02, which is higher than the normal range (18.5–24), indicating that the body is overweight. 8.78 and 38.92% of the elderly individuals have disabled and chronic issues, respectively. The household income of the elderly was 76,409.81 Chinese Yuan (CNY), which was 10.74 after being logged. For the 61–75 age group, there were a total of 1987 samples, with males accounting for 53.45%. The education level of this group is lower. 68.50% had an educational level of primary school and below, 23.86% had an educational level of junior high school, 7.50% had an educational level of high school/technical secondary school/vocal high school, and 0.15% had an educational level of college/undergraduate. 88.83% of people are married. The average BMI index is 23.50, indicating a normal body type. The household income of this elderly group was lower than the first one (63,195.57 CNY for average, and 10.46 after being logged).

**Table 1:** General characteristics of the study population (*N* = 5803)

Age range		[45, 61] (*N* = 3816)	[61, 75] (*N* = 1987)	Pearson Chi-square test	
Variable	Description			χ^2^	*P*
Consciously controlling sugar intake (N, %)	Do you consciously control sugar intake? Yes = 1, No = 0	2252(59.01%)	1228(61.80%)	4.2278	0.040
Consciously controlling salt intake (N, %)	Do you consciously control salt intake? Yes = 1, No = 0	2350(61.58%)	1233(62.05%)	0.1225	NS
Consciously controlling edible oil intake (N, %)	Do you consciously control the intake of edible oil? Yes = 1, No = 0	2230(58.44%)	1172(58.98%)	0.1601	NS
Learning health knowledge or wellness knowledge (N, %)	Do you consciously learn health or wellness knowledge? Yes = 1, No = 0	1660(43.55%)	795(40.03%)	6.6136	0.010
Gender (N, %)	Female = 0, Female = 1	1934(50.68%)	1062(53.45%)	4.0031	0.045
Education level (N, %)	Primary school and below = 1	1555(40.75%)	1361(68.50%)	417.899	0.000
	Junior high school = 2	1849(48.45%)	474(23.86%)		
	High school/technical secondary school/vocational high school = 3	376(9.85%)	149(7.50%)		
	College/undergraduate = 4	36(0.94%)	3(0.15%)		
Marriage (N, %)	Married or not, yes = 1, no = 0	3655(95.78%)	1765(88.83%)	102.488	0.000
BMI (Mean)	Weight (kg)/Height ^2^ (m)	26.02	23.50	–	–
Disabled (N, %)	Disabled or not, yes = 1, no = 0	335(8.78%)	194(9.76%)	1.5290	NS
Chronic diseases (N, %)	Whether suffering from chronic diseases, yes = 1, no = 0	1485(38.92%)	957(48.16%)	45.850	0.000
Household income (Mean, Logarithmic)	Questionnaire provides total of 8 categorie of income, using logarithms	76,409.81(10.74)	63,195.57(10.46)	–	–

*NS* No significance

### Empirical methodology

According to the New Rural Pension Scheme and Urban and Rural Resident Pension Scheme, the pension can be obtained on a monthly basis for the elderly with registered residence who have reached the age of 60 and have not accessed the Urban Workers’ Social Insurance. Therefore, we regarded only those who have reached the age of 60 to receive pension as a “quasi natural” experiment, and uses a regression discontinuity design (RDD) to evaluate the impact of the New Rural Pension Scheme on the health behavior of elderly rural residents. Previous studies have also pointed out that in rural China, there are no other policies that use the age of 60 as a boundary [44]. Therefore, the effect of the cutoff point of 60 will only be the income impact brought by the New Rural Pension Scheme, and will not be confused by other policy effects.

The regression discontinuity method can not only more accurately estimate the results caused by policy shocks, but also control the estimation bias caused by endogeneity issues. It is a causal identification method that is closer to random trials than the instrumental variable method and the double difference method [45, 46]. Regression discontinuity is divided into Sharp RD (SRD) and Fuzzy RD (FRD). If there is a deterministic change from 0 to 1 in the disposal state around the discontinuity of the driving variable, it is an accurate regression discontinuity; if the disposal variable is only a probability jump around the discontinuity, then it is a fuzzy regression discontinuity. Due to policy differences, insured individuals over 60 years old may not necessarily receive the pension, and a small number of insured individuals under 60 years old have also received the pension [47]. Therefore, this article has adopted a fuzzy discontinuity approach. The disposal variable is defined as whether an individual receives the pension, which is equal to 1 (received) or 0 (not received). The driving variable age is set to *z*_*i*_, and the outcome variable health behavior is set to *HB*_*i*_ (see eq. 1). The disposal effect estimated by the fuzzy discontinuity is the ratio of health behavior to the jump in age of the pension. Due to the emphasis on parameter estimation near the critical point in regression discontinuity, A non parametric method is adopted to estimate the numerator and denominator of eq. 1 in specific operations. We use Stata16.0 software in data processing and empirical analysis.

1 $${\uptau}_{\textrm{FRD}}=\textrm{E}\left[{\textrm{HB}}_{\textrm{i}}(1)-{\textrm{HB}}_{\textrm{i}}(0)|{\textrm{z}}_{\textrm{i}}=60\right]=\frac{\Delta \textrm{HB}}{\Delta \textrm{P}}=\frac{\underset{\upvarepsilon \to {0}^{+}}{\lim}\textrm{E}\left[{\textrm{HB}}_{\textrm{i}}|{\textrm{z}}_{\textrm{i}}=60+\upvarepsilon \right]-\underset{\upvarepsilon \to {0}^{-}}{\lim}\textrm{E}\left[{\textrm{HB}}_{\textrm{i}}|{\textrm{z}}_{\textrm{i}}=60+\upvarepsilon \right]}{\underset{\upvarepsilon \to {0}^{+}}{\lim}\textrm{E}\left[{\textrm{D}}_{\textrm{i}}|{\textrm{z}}_{\textrm{i}}=60+\upvarepsilon \right]-\underset{\upvarepsilon \to {0}^{-}}{\lim}\textrm{E}\left[{\textrm{D}}_{\textrm{i}}|{\textrm{z}}_{\textrm{i}}=60+\upvarepsilon \right]}$$

## Results

### Graphic analysis

**Fig. 1:**
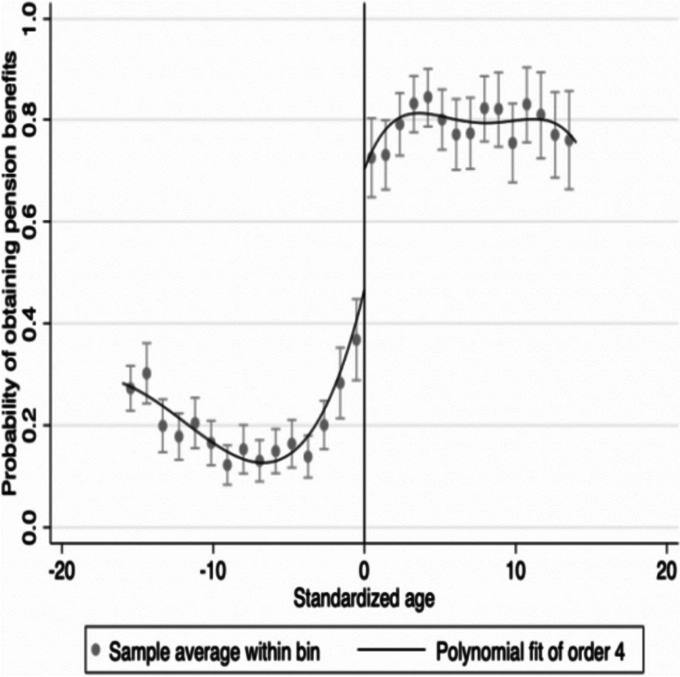
Age and probability of having access to pension

Before entering the regression analysis, this article visually displayed the relationship between driving variables and disposal variables, and the relationship between driving variables and outcome variables in the form of a graph. Displaying the relationship between driving variables and disposal variables, and the relationship between driving variables and outcome variables, has become a standard practice in regression discontinuity (RD) analysis, which helps to intuitively understand the meaning of RD [45]. Figure 1 shows the relationship between the age of the driving variable and whether to obtain the pension (the standardized age point 0 in the figure is 61 years old). It can be intuitively seen that the probability of receiving a pension has a significant jump around the standardized age of 0, which confirms that Chinese insured individuals over the age of 60 can have access to pension.

**Fig. 2:**
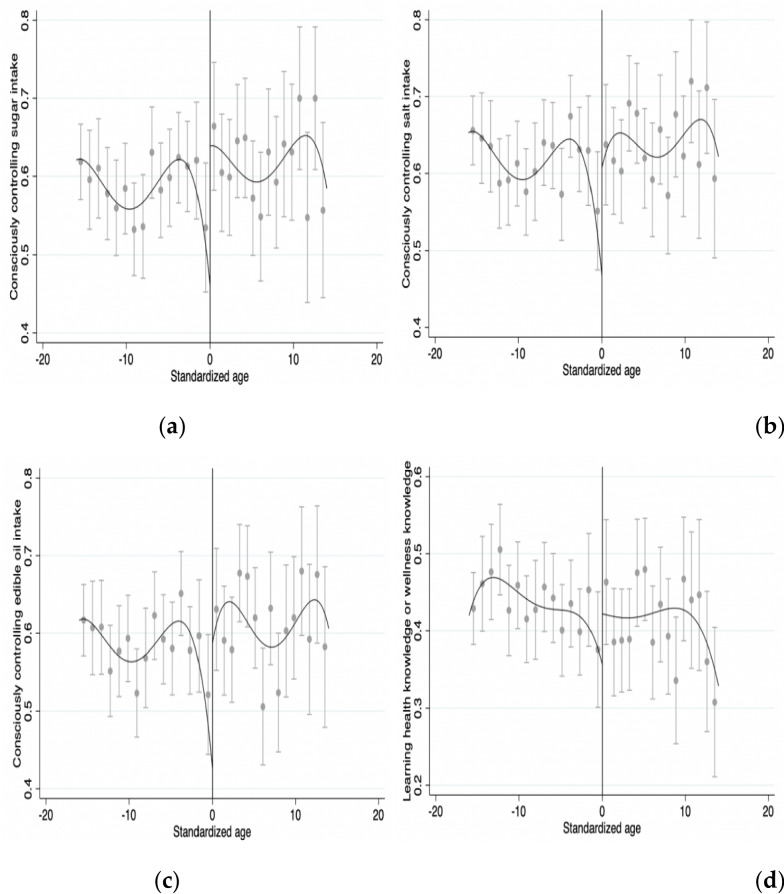
The relationship between age and controlling intake of sugar, salt and edible oil, as well as learning health or wellness knowledge

Figure 2 shows the estimated results of the principal regression, (a), (b), (c), and (d) represent the relationship between age as the driving variable and conscious control of sugar, salt, and edible oil intake, as ell as learning health or wellness knowledge. It can be intuitively seen that sugar, salt, and edible oil intake, as well as learning health or wellness knowledge, have a significant upward jump on the right side of the discontinuity, Based on Fig. 1, it can be preliminarily explained that the positive income impact brought by pension can improve the health behavior of rural residents to a certain extent.

### Regression results

We calculated the optimal bandwidth under the minimum mean square error using the method commonly used in existing research and estimates the regression results of fuzzy discontinuity. According to Panel A in Table 2, when the dependent variable is sugar intake control, the optimal bandwidth is 3.59 years, which includes 589 samples on the left side of the discontinuity and 642 samples on the right side of the discontinuity. The regression discontinuity treatment had a significantly positive effect (*P* value of 0.064, not significantly different from the 5% significance level). Panel B in Table 2 indicates that when the dependent variable is controlled salt intake, the optimal bandwidth is 3.62 years, with 589 samples entering the bandwidth on the left side of the discontinuity and 642 samples on the right side of the discontinuity. The regression discontinuity treatment effect is significantly positive at the 10% level. Panel C and Panel D in Table 2 indicate that when the dependent variable is controlling edible oil intake, the optimal bandwidth is 3.70 years, which includes 589 and 642 samples on the left and right sides of the discontinuity, respectively. When the dependent variable is learning health or wellness knowledge, the optimal bandwidth is 4.00, and the left and right samples on the discontinuity are 864 and 795, respectively. Although the effect of regression discontinuity processing is not significant, the coefficient is still positive. It can be seen that the income impact brought by pension can effectively improve the health behavior of rural residents. In addition, the first stage results in Table 2 report the impact of the driving variable age on pension receiving, with significant results at the 1% level, fully indicating that the age discontinuity can be used as a “instrumental variable” for determining whether to receive pension.

**Table 2:** Regression discontinuity estimation results

	Panel A		Panel B	
	Phase 1	Consciously controlling sugar intake	Phase 1	Consciously controlling salt intake
Pension	0.263^***^	0.561^*^	0.262^***^	0.487^*^
	(0.074)	(0.303)	(0.074)	(0.288)
Bandwidth		3.590		3.623
Control variables	Yes	Yes	Yes	Yes
N		589;642		589;642
	Panel C		Panel D	
	Phase 1	Consciously controlling edible oil intake	Phase 1	Learning health knowledge or wellness knowledge
Pension	0.264^***^	0.470	0.263^***^	0.251
	(0.074)	(0.298)	(0.073)	(0.279)
Bandwidth		3.700		4.00
Control variables	Yes	Yes	Yes	Yes
N		589;642		864;795

The robust standard error of clustering to the township level is reported in parentheses, with * *p* < 0.1, * * *p* < 0.05, * * * *p* < 0.01. The covariates include gender, education level, marital status, BMI, chronic disease, disability, and family income. The default minimum mean square error bandwidth is used in the table

### Robustness test

In order to ensure the credibility of the above conclusions, this study has done a series of robustness tests, including continuity test, different bandwidth analysis and placebo test.

### Continuity inspection

**Fig. 3:**
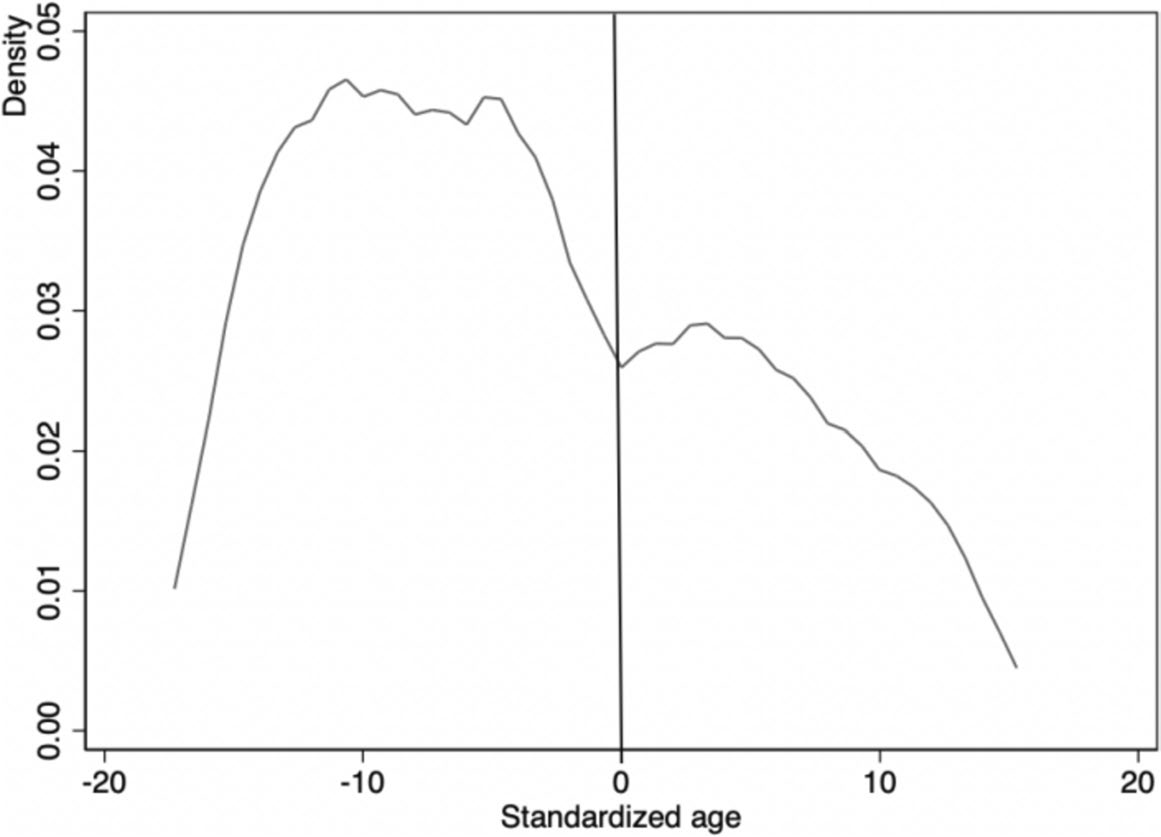
Age density distribution of the elderly population with agricultural household registration participating in resident insurance

Continuity testing is an important prerequisite for obtaining consistent estimates in regression discontinuity. This study adopts two testing methods. Firstly, it tests whether each antecedent variable is continuous at the discontinuity. The regression setting is the same as the previous text, but the dependent variable is replaced with the antecedent variable. As shown in Table 3 (1)–(7), the estimated results are not significant, indicating that all the predetermined variables have no discontinuity at the discontinuity. The second method of testing the continuity hypothesis is to observe the distribution of driving variables to test whether age is manipulated. If there is data heaping at the discontinuity, it will affect the regression discontinuity results [48]. Figure 3 shows that there is no data accumulation at the age of 61 at the discontinuity, so it can be considered that there is no discontinuity at the discontinuity and no trace of manipulation. Therefore, based on the above two test results, it can be seen that the continuity assumption of regression discontinuity is satisfied.

**Table 3:** Continuity test

	Variable	Pension	Bandwidth	N
(1)	Gender	0.032	4.315	863;796
		(0.215)		
(2)	Education level	−0.312	3.069	589;642
		(0.472)		
(3)	Marriage	−0.055	3.507	589;642
		(0.144)		
(4)	BMI	6.655	2.454	310;470
		(7.114)		
(5)	Suffering from chronic diseases	0.140	4.334	863;796
		(0.233)		
(6)	Disabled	0.102	3.743	589;642
		(0.139)		
(7)	Household income (logarithmic)	0.517	3.856	589;642
		(0.690)		

* *p* < 0.1, * * *p* < 0.05, * * * *p* < 0.01. The default minimum mean square error bandwidth is used in the table

### Different bandwidth

The optimal bandwidth used in the benchmark regression results shown in Table 2 is symmetric about both sides of the discontinuity, and Table 4 uses different bandwidth on both sides for estimation as a robustness test. As shown in Table 4, when the dependent variable is controlling sugar intake, the left bandwidth of the discontinuity is 3.516, which accommodates 589 samples, and the right bandwidth of the discontinuity is 5.839, which accommodates 976 samples. The regression discontinuity results are significant at the 10% level. When the dependent variable is controlling salt intake, the left bandwidth of the discontinuity is 3.482, which accommodates 589 samples, and the right bandwidth is 6.175, which accommodates 1120 samples. Although the regression results are not significant, the *p*-value is 0.105, which is close to the 10% significance level. In addition, this article also customized 3.5 years and 4.5 years as bandwidth, and repeated regression discontinuity. When the dependent variable was controlled for sugar intake, both had a significance level of 10%; When the dependent variable is controlling salt intake, the bandwidth is positively significant at the 10% level for 3.5 years. Although the bandwidth is not significant at 4.5 years, the p-value is 0.106, which can be considered close to the 10% significance level and the coefficient is also positive. In summary, the regression results under different bandwidths can demonstrate that the pension income can improve the health behavior of rural residents.

**Table 4:** Estimation results under different bandwidths

	Panel A			Panel B		
	Consciously controlling sugar intake			Consciously controlling salt intake		
Pension	0.517^*^ (0.288)	0.570^*^ (0.313)	0.464^*^ (0.252)	0.434 (0.267)	0.487^*^ (0.295)	0.387 (0.239)
Bandwidth	3.516;5.839	3.5	4.5	3.482;6.175	3.5	4.5
Control variables	Yes	Yes	Yes	Yes	Yes	Yes
N	589;976	589;642	863;796	589;1120	589;642	863;796

* *p* < 0.1, * * *p* < 0.05, * * * *p* < 0.01; Consciously controlling edible oil intake and learning health or wellness knowledge are not significant in the second stage of Table 2, therefore different bandwidth estimation results are not shown here and can be obtained from the author

### Placebo test

Drawing inspiration from the previous using placebo testing, to demonstrate that health behavior is only affected by the income shock of pension funds, rather than other mechanisms, then making “pseudo discontinuity” at other ages away from 60 years old should not result in significant regression results. This article also selected 56, 57, 58, 62, 63, and 64 years old as “pseudo discontinuity” for placebo testing. As shown in Panel A and Panel B in Table 5, the regression results are not significant, indicating that the improvement of health behavior near the age of 60 is indeed the effect of the pension benefits.

**Table 5:** Placebo test

	(1)	(2)	(3)	(4)	(5)	(6)
Discontinuity	56	57	58	62	63	64
	Panel A (Dependent variable: consciously controlling sugar intake)					
Pension	−4.962	−0.978	−0.416	0.439	0.056	6.246
	(20.453)	(1.672)	(1.465)	(0.357)	(0.584)	(27.588)
Bandwidth	2.898	3.818	4.783	3.460	4.577	3.898
N	529;797	773; 863	1047;887	441;665	608;822	470;650
	Panel B (Dependent variable: consciously controlling salt intake)					
Pension	0.675	−2.320	−1.412	0.198	0.270	8.830
	(6.096)	(2.342)	(1.214)	(0.327)	(0.612)	(36.632)
Bandwidth	2.992	4.110	3.168	3.336	4.566	3.967
N	529;797	995;994	779;720	441;665	608;822	470;650

The robust standard error of clustering to the township level is reported in parentheses, with * *p* < 0.1, * * *p* < 0.05, * * * *p* < 0.01. The default minimum mean square error bandwidth is used in the table

### Heterogeneity analysis

We selected income and gender for further heterogeneity analysis.

### Income level

The difference in economic level is an important reason for the difference in farmers’ health [49]. Table 6 objectively selected household income and subjectively selected household income satisfaction as measurement indicators for income heterogeneity analysis. In terms of household income, according to the three-way method, the sample is divided into low-income, middle-income, and high-income. The regression discontinuity effects of Panel A and Panel B both show that low-income families are more likely to improve their health behavior under the impact of receiving pension benefits. In terms of household income satisfaction, based on the question “Are you satisfied with your family’s current income level?”, the sample is divided into satisfied, commonly, and dissatisfied. The results of Panel A and Panel B show that rural residents who believe that their household income is average are more likely to improve their health behavior after receiving pension benefits. From the coefficient perspective, The coefficient of health behavior of rural residents who are dissatisfied with their family income under the impact of the new rural social security pension is relatively equal to or higher than the coefficient of satisfaction with their family income (Panel A) or higher (Panel B). The regression effects of Panel C and Panel D, although not statistically significant, still have a similar distribution as the coefficients of Panel A and Panel B. The above results indicate that the exogenous impact of the pension income is more likely to improve the health behavior of medium and low income rural residents, and therefore has positive policy implications.

**Table 6:** Heterogeneity analysis: income level

Income level		Panel A:Dependent variable: consciously controlling sugar intake			Panel B:Dependent variable: consciously controlling salt intake		
		Pension	Optimal bandwidth	N	Pension	Optimal bandwidth	N
(1) Household income	Low-income	0.630^**^	4.156	293;324	0.451^*^	4.458	293;324
		(0.287)			(0.266)		
	Medium-income	0.059	5.734	373;307	0.170	5.717	373;307
		(0.312)			(0.329)		
	High-income	0.811	4.499	291;219	0.488	4.990	291;219
		(1.643)			(1.169)		
(2) Income satisfaction	Satisfied	0.113	4.336	476;471	0.038	4.284	476;471
		(0.401)			(0.399)		
	Commonly	0.622*	5.210	241;205	0.538*	5.175	241;205
		(0.323)			(0.318)		
	Dissatisfied	1.026	3.700	136;134	1.198	3.358	136;134
		(0.899)			(1.021)		
Income level		Panel C:Dependent variable-Consciously controlling edible oil intake			Panel D:Dependent variable-Learning health knowledge or wellness knowledge		
		Pension	Optimal bandwidth	N	Pension	Optimal bandwidth	N
(1) Household income	Low-income	0.410	4.267	293;324	0.264	4.569	293;323
		(0.264)			(0.241)		
	Medium-income	0.518	5.404	373;307	0.493	5.096	373;307
		(0.416)			(0.417)		
	High-income	0.218	4.973	291;219	−3.146	2.871	101;136
		(1.166)			(23.737)		
(2) Income satisfaction	Satisfied	0.132	4.530	476;471	0.065	4.230	476;470
		(0.401)			(0.424)		
	Commonly	0.409	5.170	241;205	0.422	4.994	188;159
		(0.326)			(0.321)		
	Dissatisfied	1.052	4.229	199;166	0.155	4.965	199;166
		(0.766)			(0.476)		

The robust standard error of clustering to the township level is reported in parentheses, with * *p* < 0.1, * * *p* < 0.05, * * * *p* < 0.01. The default minimum mean square error bandwidth is used in the table

### Gender

As shown in Panel A, Panel B, and Panel C in Table 7, when the dependent variable is controlling for sugar, salt, and edible oil intake, the regression discontinuity effect of the male rural resident sample is significantly positive at the 5% level, while it is not significant for women. When the dependent variable is learning health knowledge or wellness knowledge (Panel D), although the regression discontinuity effect is not significant for both men and women, the coefficient of the male sample is still positive. The above results indicate that the new rural pension income has a more significant improvement effect on the health behavior of male rural residents.

**Table 7:** Heterogeneity analysis: gender

Gender	Panel A:Dependent variable-consciously controlling sugar intake			Panel B:Dependent variable-consciously controlling salt intake		
	Pension	Optimal bandwidth	N	Pension	Optimal bandwidth	N
Female	1.413	3.628	278;305	−0.722	3.675	278;305
	(8.494)			(6.253)		
Male	0.391^**^	4.511	453;428	0.390^**^	4.449	453;428
	(0.168)			(0.166)		
Gender	Panel C:Dependent variable-Consciously controlling edible oil intake			Panel D:Dependent variable-Learning health knowledge or wellness knowledge		
	Pension	Optimal bandwidth	N	Pension	Optimal bandwidth	N
Female	0.741	3.565	278;305	− 157.642	4.541	410;367
	(5.361)			(26,222.371)		
Male	0.378^**^	4.273	453;428	0.021	3.361	311;337
	(0.179)			(0.215)		

The robust standard error of clustering to the township level is reported in parentheses, with * *p* < 0.1, * * *p* < 0.05, * * * *p* < 0.01. The default minimum mean square error bandwidth is used in the table

## Discussion

This study uses data from China Rural Revitalization Survey in 2020 and uses fuzzy discontinuity regression method to investigate the relationship between pension receipt and health behavior of Chinese older rural residents. The results indicate that pension significantly improved the health behavior of rural older adults. Specifically, pension can significantly improve the probability of rural older residents’ conscious control of sugar and salt intake, which is still valid after a series of robustness tests such as continuity test, different bandwidth analyze, and implementation of placebo test. In addition, when the outcome variable is conscious control of edible oil, learning health knowledge, or health preservation knowledge, although the regression discontinuity treatment effect is not significant, the coefficient is still positive, thus overall confirming the improvement effect of the New Rural Pension Scheme on health behavior. Heterogeneity analysis shows that the pension benefits are more likely to improve the health behavior of rural older residents from low-income families and self rated families with average income, as well as men.

Two mechanisms can explain our findings. The first is the income effect of pension. Health will depreciate with age [50]. People often attach great importance to health preservation in order to seek improvement in their health status [51], thereby reducing harmful behaviors to their physical health, such as the nutritional and dietary behavior indicators selected in this article. Since 1978, urban areas in China have implemented a retirement age system for males aged 60 and females aged 50 or 55. Subsequently, social insurance systems such as enterprise employee pension insurance have been established to ensure retirement pension. However, as the urban and rural registered residence system has not been broken, there is no statutory retirement age in rural areas, and the elderly in rural areas have no pension. Therefore, most elderly people in rural areas still earn income by providing labor supply after reaching retirement age. The implementation of the New Rural Pension Scheme can ensure that the elderly population in rural areas receive pension after the age of 60. According to the income effect of the pension, which can smooth out expected risks, relax individual budget constraints, and motivate individuals to increase leisure time and reduce labor supply [52], older people in rural areas do not need to work, pay more attention to their physical health, and adopt healthier individual behavior.

The “spillover effect” of pension cannot be ignored. Most studies focus on the impact of China’s new agricultural insurance policy on intergenerational support and generational care [53, 54]. Intergenerational support means that under the motivation of “altruism” and “exchange and mutual assistance” with children in China, the older residents will provide financial support for their children after receiving pension, and gain more attention from their children; generational care refers to the increased care for grandchildren by rural older residents who do not need to work after receiving the pension. These two “spillover effects” brought about by pensions will make older people in rural areas pay more attention to their physical health.

Moreover, heterogeneity analysis found that pension is more likely to improve the health behavior of rural elderly residents from middle and low-income families, indicating that the marginal effect of pension is very significant. This is not only consistent with the conclusions of existing research [55], but also indicates the positive role of the New Rural Pension Scheme in promoting health equality among elderly residents in rural areas. And the possible explanation for the significant improvement in health behavior among male rural elderly residents is the influence of traditional Chinese family division, namely “men outside, women inside”. Women still need to handle a large amount of household chores and occupy leisure time [56], which affects the effectiveness of the New Rural Pension Scheme in improving health behavior.

This study contributes to the literature on the effect of pension on health behavior by providing more evidence from China. In this study, we provide more comprehensive health behavior’s indicators than previous literature, namely Chinese rural elderly residents consciously control sugar intake, salt intake, edible oil intake, and learn health knowledge. Meanwhile, we provide more accurate assessments, including the results of continuity inspection, different bandwidth analyses, placebo test and sub-sample groups.

Of course, this research is not without limitations. Due to the lack of relevant variables in the questionnaire, the mechanism of the effect of having access to pension on the health behavior of rural residents has not been tested. Another limitation is the fact that we were not able to include more detailed scale to measure the health behavior of older people under the impact of pension. For example, the types of edible oils, food and certified food consumption details and structure. The above limitations also provide direction for further improvement in subsequent research, especially for developing or underdeveloped regions.

## Conclusions

The purpose of this study is to analyze the association between pension and health behavior in rural China. Since healthy aging is essential for older adults’ quality of life and for national development, clarifying the factors or policies that promote better health behavior is of vital importance. This research confirms the promoting effect of the New Rural Pension Scheme on the health behavior of older people in rural areas in China and conducts sub-sample groups analysis, providing a scientific quantitative basis for the older social insurance system to motivate residents to improve their health behavior.

The results of this study have important implications for policy makers to further achieve healthy aging in rural areas. On the one hand, the New Rural Pension Scheme should be further improved and the pension standards should be raised. Healthy behavior is a relatively low-cost measure that can generally improve public health [57]. Research has confirmed the health promotion effect of pension income on elderly rural residents, but currently there are characteristics of low basic pension and large pension gap between urban and rural residents in rural areas. From 2013 to 2018, the gap in pension income between urban and rural elderly residents continued to widen, with urban elderly residents’ pension income being 11.3 times that of rural elderly residents in 2018 [58]. Therefore, it is necessary to continue to optimize the policy to improve the health behavior of rural residents, narrow the urban-rural health gap, and enable the entire population to share the fruits of economic development. On the other hand, it is essential to increase subsidies for impoverished rural residents and promote the development of good health behaviors. It is found that there is significant inequality in pension benefit in rural China, and pension benefit inequality is significantly higher in rural than in urban areas [59], and the study results of this article prove that for the poorer rural residents, the marginal utility of pension benefits will be greater, so it is necessary to increase their subsidies; however, at the same time, impoverished residents may often fall into the “health trap” (that is, they prefer to spend high prices for treatment rather than cheap prevention). Therefore, strengthen the promotion of good health behavior among rural residents and comprehensively leverage the policy effect of pension to reduce health inequality is equally necessary.

## Data Availability

This study used the de-identified data from the China Rural Revitalization Survey (CRRS) in 2020, which were conducted by Rural Development Institute, Chinese Academy of Social Sciences. These data are publicly available, and for research purposes only. Users need to apply in the name of an institution. Questionnaires and the datasets are available upon reasonable request and with permission of Rural Development Institute, Chinese Academy of Social Sciences. The datasets can be requested as: (a) visit to: http://rdi.cass.cn/ggl/202210/t20221024_5551642.shtml; (b) fill out the data usage application form as website’s required, and send it to the reserved email on the website. The staff will complete the review within 1 week.
